# Terbium Removal from Aqueous Solutions Using a In_2_O_3_ Nanoadsorbent and *Arthrospira platensis* Biomass

**DOI:** 10.3390/nano13192698

**Published:** 2023-10-03

**Authors:** Amal H. Al-Bagawi, Nikita Yushin, Nasser Mohammed Hosny, Islam Gomaa, Sabah Ali, Warren Christopher Boyd, Haitham Kalil, Inga Zinicovscaia

**Affiliations:** 1 Chemistry Department, Faculty of Science, University of Ha’il, Ha’il City 1560, Saudi Arabia; amalelbagawi@gmail.com; 2 Department of Nuclear Physics, Joint Institute for Nuclear Research, Joliot-Curie Str., 6, 141980 Dubna, Russia; ynik_62@mail.ru; 3 Department of Chemistry, Faculty of Science, Port Said University, Port Fouad P.O. Box 42522, Egypt; nasserh56@yahoo.com; 4 Nanotechnology Research Centre (NTRC), The British University in Egypt (BUE), Suez Desert Road, El-Sherouk City, Cairo 11837, Egypt; islam.gomaa@bue.edu.eg; 5 Department of Microbiology, Faculty of Veterinary Medicine, Cairo University, Giza 12613, Egypt; sabahali55lolo@gmail.com; 6 Chemistry Department, Cleveland State University, Cleveland, OH 44115, USA; w.c.boyd59@csuohio.edu; 7 Chemistry Department, Faculty of Science, Suez Canal University, Ismailia 41522, Egypt; 8 Department of Nuclear Physics, Horia Hulubei National Institute for R&D in Physics and Nuclear Engineering, 30 Reactorului Str. MG-6, 077125 Magurele, Romania

**Keywords:** green synthesis, In_2_O_3_, adsorption, terbium (Tb), *Arthospira platensis*, extraction, isotherm, kinetics

## Abstract

Terbium is a rare-earth element with critical importance for industry. Two adsorbents of different origin, In_2_O_3_ nanoparticles and the biological sorbent *Arthrospira platensis*, were applied for terbium removal from aqueous solutions. Several analytical techniques, including X-ray diffraction, Fourier-transform infrared spectroscopy, and scanning electron microscopy, were employed to characterize the adsorbents. The effect of time, pH, and terbium concentration on the adsorption efficiency was evaluated. For both adsorbents, adsorption efficiency was shown to be dependent on the time of interaction and the pH of the solution. Maximum removal of terbium by *Arthrospira platensis* was attained at pH 3.0 and by In_2_O_3_ at pH 4.0–7.0, both after 3 min of interaction. Several equilibrium (Langmuir, Freundlich, and Temkin) and kinetics (pseudo-first order, pseudo-second order, and Elovich) models were applied to describe the adsorption. The maximum adsorption capacity was calculated from the Langmuir model as 212 mg/g for *Arthrospira platensis* and 94.7 mg/g for the In_2_O_3_ nanoadsorbent. The studied adsorbents can be regarded as potential candidates for terbium recovery from wastewater.

## 1. Introduction

The rare-earth elements (REEs) are a group of 15 elements of the lanthanide series as well as scandium and yttrium. REEs possess very similar chemical properties, while distinctive electromagnetic, catalytic, and optical capabilities make them crucial resources for the production and development of high-technology products [1,2]. The unique properties of REEs allow them to be considered “industrial vitamins”. Terbium (Tb), along with dysprosium, europium, neodymium, and yttrium, amount for approximately 85% of the total use of REEs in final products [3,4,5]. Terbium is a soft REE widely applied in the production of components for smartphones, laptops, sensors, and televisions, and it is also an important constituent of magnets, microphones, etc. Moreover, terbium is a key component in fluorescent lamps [6,7]. Terbium, together with ZrO_2_, is employed as a crystal stabilizer in fuel cells used at high temperatures, as well as a dopant for solid-state materials such as calcium fluoride, calcium tungstate, and strontium molybdate [8,9]. 

Concomitant with the growing demand for terbium by industrial enterprises, the amount of electronic waste containing this element has significantly increased. Moreover, mining, unregulated waste disposal, and the absence of proper recycling processes contribute to rather high level of REEs in water effluents, ranging from 1 to 200 mg/L [5,10]. It has been reported that the concentration of terbium in sediments from the Baram River in Malaysia was between 0.68 and 0.8 mg/kg [11]. Other reports quantifying the concentration of this metal showed a maximum of 1.8 mg/L in the water of the Atibaia River in Brazil [12], 1.13 mg/kg in the sediments of the Ipojuca River in Brazil [13], and a maximum of 2.6 mg/kg in the Lubumbashi River near an old closed mine in the Democratic Republic of the Congo [14]. 

The toxicity of terbium is a concern, although data related to its harmful effects are still scarce. Nevertheless, several papers have mentioned its negative impact on humans, plants, animals, aquaculture, and the wider ecosystem. Shimida et al. [15] reported that single parenteral injections of 20 or 200 μmol TbCl_3_/kg in mice caused increased pulmonary weight, rapid lipid peroxidation, and an elevated protein content. Also, juvenile rainbow trout exposed to terbium or praseodymium for 96 h showed LC_50_ of 5.8 and 11 mg/L, respectively. In addition to acute toxicity, terbium also exhibited DNA repair activity, inflammation, protein denaturation, calcium binding, and oxidative stress [16]. Ecotoxicological responses to terbium were reported to involve loss of redox balance and neurotoxicity, as well as metabolic impairment, upon exposure to Tb in clams, which are considered a common seafood in many countries [17]. Studies have also shown phytotoxic effects of terbium in horseradish roots and leaves [18,19].

Consequently, it is crucial to find effective, eco-friendly, and cheap techniques for the recovery of terbium ions. Well-established technologies used to remove REEs from solutions include chemical precipitation, filtration, solvent extraction, ion exchange, membrane technology, and adsorption [20,21,22]. However, traditional methods applied for wastewater treatment are considered inefficient in cases of diluted effluents, which require energy, chemicals, and significant costs, while some may even produce secondary toxic pollutants [3,23,24]. Adsorption is one of the most popular techniques applied for metal removal due to its simplicity, low cost, ability to treat diluted metal solutions and/or unclarified solutions, high adsorption capacity, and adsorbents regeneration [25,26,27]. Moreover, materials produced from agricultural or biotechnological waste can be used as sorbents [3,21,22,24], along with commercial adsorbents. The question of which adsorbents (biological or specially synthesized) are more suitable for large-scale industrial application often arises.

In the present study, the adsorption capacity of two adsorbents, the cyanobacterium *Arthrospira platensis* and In_2_O_3_ nanoparticles (NPs), toward terbium ions was assessed under different experimental conditions. 

*Arthrospira platensis* (spirulina) is a well-known cyanobacterium with high adaptability to high alkalinity, temperature, salt concentration, and different pollutants in culture media [28,29]. Other advantages of spirulina include ease of handling, high biomass productivity, and a high metal biosorption capacity [28,30,31,32,33]. Indium oxide (In_2_O_3_) is widely applied as a semiconductor in photocatalytic degradation, solar cells, and gas sensing [34,35,36,37,38]. The high surface area and low coordination number of In_2_O_3_ may make it a good adsorbent for inorganic and organic pollutants [39,40,41]. 

## 2. Materials and Methods

All chemicals were used in the experiments without further purification: indium acetate, (≥99%, Oxford, UK), crystalline anhydrous citric acid (C_6_H_8_O_7_) (≥99.5%, Fisher Chemical, Loughborough, UK), HCl, NaOH, and deionized (DI) Mille-Q water (COD ≤5 ppb). *Artrhospira platensis* CNMN-CB-02 (*A. platensis*, spirulina), used as biosorbent, was obtained from the collection of non-pathogenic microorganisms (IMB TU, Chisinau, Moldova). The process of biomass growth is described in detail in the literature [29]. After cultivation for six days, the biomass was separated from the medium, dried, and homogenized for 10 min in a planetary ball mill (PULVERISETTE 6, Fritsch Laboratory Instruments GmbH, Idar-Oberstein, Germany) at 400 rpm.

### 2.1. Synthesis of Precursor of In_2_O_3_ NPs

The precursor for In_2_O_3_-NPs synthesis was prepared via a green solvothermal method. Indium acetate powder was mixed with citric acid in equimolar amounts in mortar to obtain a very fine powder, and then 1 mL of Mille-Q-water was added under continuous grinding until the appearance of an acetic acid odor, a change in form (paste), and a yellowish color. The obtained paste was solvated in 100 mL of Mille-Q-water and stirred for 2 h at 400 rpm, then irradiated for 30 min via probe sonication (20 kHz) in pulsed mode, and finally dried under vacuum at 100 °C overnight. The resulting transparent sheets are the precursor. In the next stage, the obtained precursor powder was calcinated at 700 °C for 2 h under inert conditions (nitrogen flow) with a temperature gradient rate of 5 °C per minute. The obtained powder was characterized as In_2_O_3_-nanoparticels (In_2_O_3_-NPs) as described in the schematic synthesis diagram Figure S1.

### 2.2. Adsorption Experiments

To prepare the terbium solutions, Tb(NO_3_)_3_·6H_2_O (Sigma Aldrich, Darmstadt, Germany) was dissolved in distillated water. Experiments were carried out in Erlenmeyer flasks of 50 mL volume, where 20 mL of terbium solution with a Tb concentration of 10 mg/L was mixed with 0.1 g of In_2_O_3_-NPs or spirulina biomass. To assess the effect of the activity on terbium removal, the solutions with different pHs ranging from 2.0 to 7.0 were prepared using 0.1 M HCl or NaOH. Kinetics experiments were performed, varying the time of reaction from 1 to 120 min, while maintaining other parameters constant. Adsorption equilibrium was investigated at terbium concentrations of 10–100 mg/L, while other experimental conditions were constant. All experiments were performed in triplicate.

The adsorption capacity (q) and terbium removal efficiency (E) were computed from Equations (1) and (2):

(1) $$q=\frac{V\left(C_{i}-C_{f}\right)}{m}$$

(2) $$E=\frac{C_{i}-C_{f}}{C_{i}}\cdot 100\%$$

where q is the content of terbium adsorbed, mg/g; V is the volume of solution, mL; C_i_ and C_f_ are initial and final terbium concentrations in the solution, mg/L; and m is sorbent dosage, g.

### 2.3. Characterization

The UV-Vis absorption spectra of the In_2_O_3_-NPs samples were measured using a double-beam spectrophotometer (Cary 5000 UV-Vis-NIR, Agilent Technologies, Santa Clara, CA, USA). The FTIR spectra for both adsorbents before and after terbium adsorption were collected using a FTIR spectrometer (Vertex 70, Bruker, Germany); the spectra were recorded in a spectral range of 4000–400 cm^−1^ with a spectral resolution of 3 cm^−1^. The X-ray diffraction (XRD) data were obtained using a Malvern Panalytical Empyrean 3 diffractometer to determine the phase composition and crystal structure of precursors and In_2_O_3_-NPs. The morphology and particle size of the samples were characterized using field-emission scanning electron microscopy (FESEM, Quattro S, Thermo Scientific, Waltham, MA, USA). An ICP-OES PlasmaQuant PQ 9000 Elite spectrometer (Analytik Jena, Jena, Germany) was used to determine the initial and final concentrations of terbium in experimental solutions. Zeta potential results were determined on a Malvern zeta potential and particle size analyzer (Zeta sizer Ver. 7.12). They are presented as the mean of many repeated and automated scans (12 cycles). The raw data of measurements are given in Supplementary File S1.

### 2.4. Statistics

All experiments were performed in triplicate, and values are presented as the mean of three experiments ± standard deviation. To elucidate the difference between experimental and initial values, Student’s *t*-test was applied.

## 3. Results

### 3.1. Adsorbents Characterization

**Figure 1:**
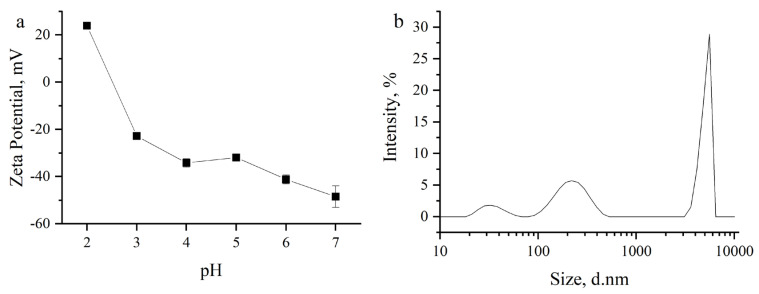
(**a**) Zeta potential and (**b**) particle size distribution of spirulina biomass.

A detailed characterization of *A. platensis* is provided in [29]. In Figure 1, the particle size distribution (Figure 1a) and zeta potential (Figure 1b) of spirulina biomass at different pH values are presented. At pH 2, the zeta potential was positive at 23.3 mV, suggesting a positive charge of the spirulina biomass surface. At pH ranges of 3–6, the values of the zeta potentials were negative, varying from −22.2 to −45.2 mV, indicating the negative charge of the biomass surface. The size of the main part of the biomass particles was in the range of 90–300 nm and 3.5–5.5 µm.

**Figure 2:**
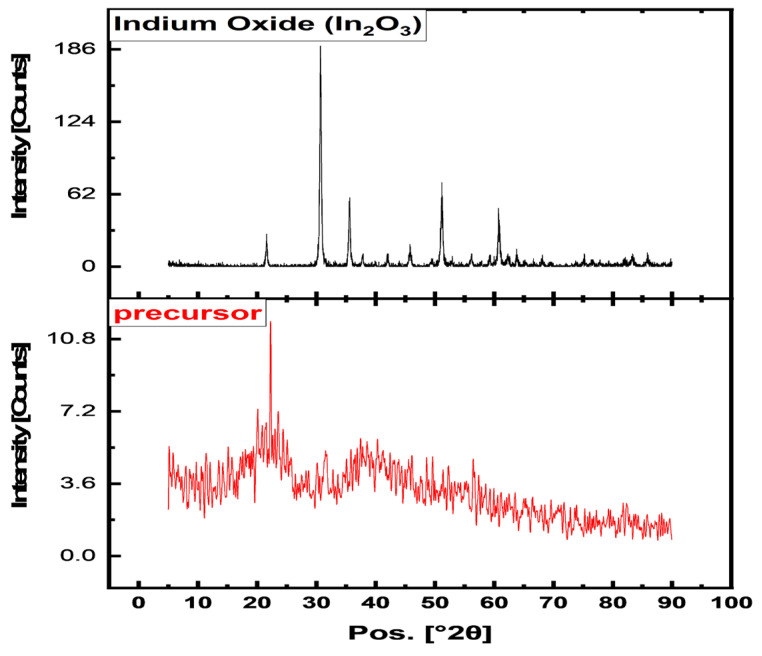
XRD pattern of In_2_O_3_-NPs and precursor.

The In_2_O_3_-NPs characterization is described below. Application of XRD for biological adsorbents characterization showed that the broad peak around 2θ = 20° corresponds to the amorphous phase of biomass [42]. The XRD pattern of In_2_O_3_ (Figure 2) shows that In_2_O_3_ NPs have a cubic crystal structure, which typically exhibits diffraction peaks at 2θ values of approximately 30.6°, 35.6°, 51.7°, 60.7°, and 83.4°, corresponding to the (222), (400), (440), (622), and (662) planes, respectively [36]. Full d-spacing, calculated using the HighScore Plus software 5.1, is shown in Figure S2 and Table S1. All the diffraction peaks in the sample (Figure 1) could be attributed to In_2_O_3_ (JCPDS No. 06-0416), as shown in Figure S2. It is worth noting that after calcination, the diffraction peaks became much higher and sharper compared with the precursor diffraction pattern, while their positions completely changed. Figure 2b shows the diffraction pattern of the semi-crystalline nature of the precursor accompanied by featured positions of indium hydroxide, In(OH)_3_, and remaining unbound hydrated citric acid correspond to different reference standard cards, such as: JCPDS: 00-004-0182 and many JCPDS, which demonstrate the crystalline nature of precursor salt (Figure S3). There were no obvious impurity peaks in the XRD pattern of In_2_O_3_ NPs, and thus the obtained yellow powder is high-purity In_2_O_3_ of cubic structure. The indium oxide crystal structure is cubic bixbyite (space group Ia-3), which consists of a face-centered cubic lattice of oxygen atoms with indium atoms occupying half of the tetrahedral sites. It is worth mentioning that (222) surface is mainly oxygen-terminated, giving the constituent indium atoms of In_2_O_3_ a low coordination number and this good indication for reactivity and good adsorption capacity [38,39]. The average crystallite size of the In_2_O_3_ NPs was calculated using the well-known Scherer equation [43] (Equation (3)).where is k is a shape constant, λ is the wavelength of the X-ray beam, β_D_ is the full width at half maximum in radians and θ is the angle of diffraction in radians.

Using Equations (4) and (5), the dislocation density (δ) and micro-strain (ε) were obtained [44].

(3) $$D\;\left(\mathrm{crystallite}\;\mathrm{size}\right)\;\mathrm{in}\;\mathrm{nm}=\left[\frac{\left(k\right)\times \left(\lambda \right)}{\left(\beta_{D}\right)\times \left(\mathrm{Cos}\theta \right)}\right]$$

(4) δ=1D2

(5) ε=β cosθ\4

The crystallite size was estimated using the full width at half maximum (FWHM) of the most intense peaks. The calculated particle size was estimated and found to be 0.743 nm and 28 nm for the In-precursor and In_2_O_3_-NPs, respectively. All the estimated parameters are shown in Table 1. The high values of the dislocation densities were obtained for semi-crystalline precursors with a remarkable decrease in the case of high-crystalline In_2_O_3_-NPs after calcination. This can be explained by the introduction of a crystallographic defect in the microstructure related to the particle size and crystallinity of structure. For In_2_O_3,_ a dislocation density of 0.00122 indicates that the material has a relatively very low concentration of dislocations. This can be beneficial for certain applications, as lower dislocation densities can lead to improved mechanical and electrical properties, such as higher strength, hardness, and electrical conductivity [45].

**Figure 3:**
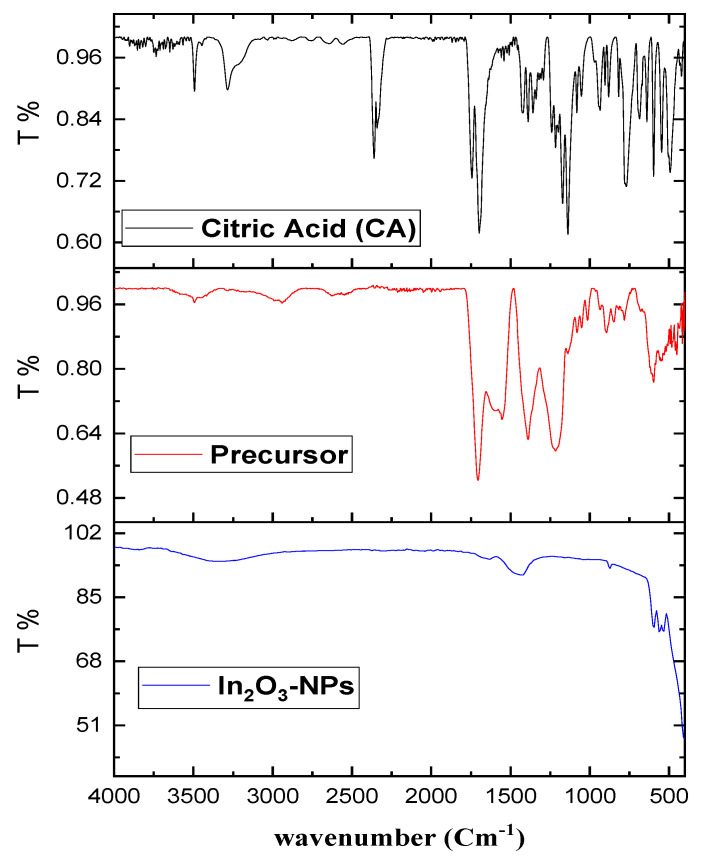
FT-IR spectra of citric acid as ligand, precursor, and In_2_O_3_-NPs.

The IR spectrum of the precursor (Figure 3) was compared with that of the free citric acid. Citric acid has three carboxyl groups; two of them are symmetric, and the third exists in a different electronic environment; hence, the citric acid spectrum contains two bands at 3500 and 3300 cm^−1^ due to the ν (OH) groups. Because of the free (OH), another shoulder band appears at 3228 cm^−1^. In addition to that, two strong bands are observed at 1742 and 1700 cm^−1^ owing to the ν_as_- (COOH) of the protonated three carboxyl groups [46]. On the other hand, the spectrum of the precursor exhibits shifts in the bands of ν (OH) of the two carboxyl groups to 3489 cm^−1^. The band of the free (OH) group has disappeared as a result of coordination to In^3+^. A noticeable change in the intensity of the band of the protonated carboxyl group ν(COOH) and a shift of the band at 1697 cm^−1^ attributed to the ν (COO^−^) of the deprotonated carboxyl group are observed. The difference between the asymmetric and symmetric (at 1400 cm^−1^) carboxylate group is 180 cm^−1^, indicating a mono-dentate character of this group. Two new weak bands are noticed at 625 and 522 cm^−1^ owing to In-O. It is suggested that citric acid chelates In (III) via carboxylate oxygen and the deprotonated hydroxo oxygen, forming a coordination compound. The FTIR spectrum of In_2_O_3_-NPs shows characteristic absorption bands in the range between 400 and 550 cm^–1^. The shape, number, and wavenumber position of these bands depend on the chemical composition, morphology, and crystal structure of the materials [41,47]. The FT-IR spectrum of the nanoadsorbent will be described in Section 3.3.

**Figure 4:**
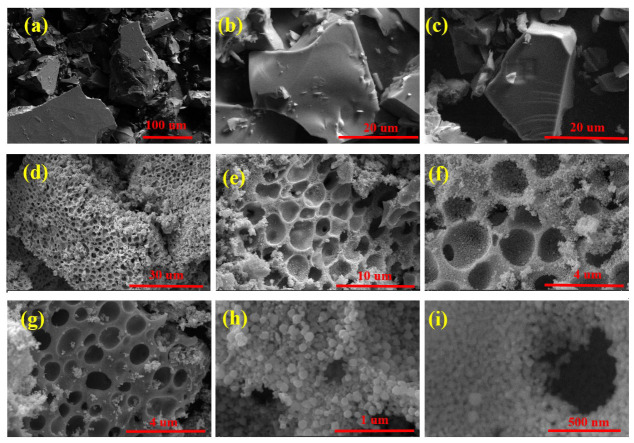
SEM images of (**a**–**c**) precursor crystallite clusters (**d**–**i**) In_2_O_3_ NPs at different magnifications.

Surface and deep insight morphology investigations show the big blocks with smoothed surfaces of the raw material used as a precursor for In_2_O_3_ nanoparticle production. In the FE-SEM of the precursor, shown in Figure 4a,c, the particles had three-dimensional growth surrounded by smooth surfaces and sharp edges in continuous interactions, represented as a connected matrix with the same nature. The FE-SEM indicates that the particles of the precursor appear to be closely packed or densely arranged, without visible spaces or voids between them. This suggests a high degree of particle packing or aggregation. Observed fragments or granules exhibit a cohesive structure, where individual particles or grains are connected to each other. However, the bonding between these particles or grains is relatively weak, indicating that they are not tightly bound together. Fragments are coated by or associated with organic compounds. These organic moieties interact with the focused electron beam, causing the particles to appear bright in the FESEM image. The brightness can be attributed to the scattering or emission of electrons from the organic molecules upon electron beam interaction [48]. After calcination at a high temperature under an inert atmosphere, with a low rate of rising temperature, the indium oxide material exhibits a specific growth pattern characterized by well-ordered and structured growth in addition to the presence of voids or channels within the material. Figure 4d–f show the observed behavior of the particles after the calcination process, indicating that the particles of indium oxide remain connected to each other, forming a continuous structure or network. This connectivity is maintained even during the gas elaboration process, which suggests that the particles have a strong tendency to adhere or bond together due to strong interparticle bonding or cohesion with each other. The calcination created constructed chips of indium oxide as building blocks with large, connected sponge-like structures with semi-circular gaps like bee hives surrounding the whole body of agglomerations. The creation of pores and holes between In_2_O_3_ NP agglomerations, as shown in Figure 4g–i, may be a result of gas evolutions during calcination, and they may be considered as attractive features for adsorption capacity and catalysis capabilities [49].

**Figure 5:**
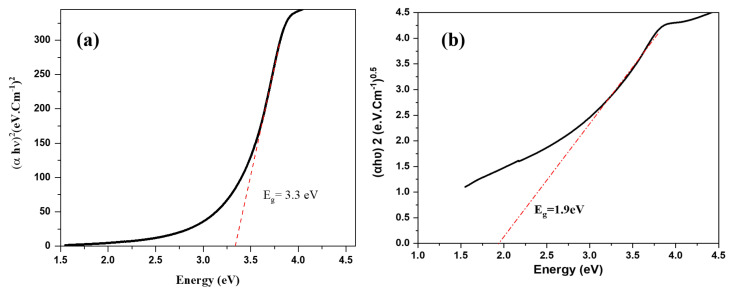
Tauc plot for indium oxide nanoparticles’ (**a**) direct band gap energy E_g_ and (**b**) indirect band gap energy (E_g_).

For interpretation of the absorption profile and optical characteristics of the NPs, UV-visible absorbance spectra are necessary. The absorption band edge of In_2_O_3_-NPs was seen in Figure 5a and corresponds to a band gap energy of 3.3 eV (Tauc plot) [50]. The synthesized In_2_O_3_-NPs also showed an indirect band gap energy (E_g_) at 1.9 eV (Figure 5b). There is a remarkable change in the maximum wavelength and a decrease in the band gap energy (3.3 and 1.9 eV) in comparison to the 3.6 and 2.2 eV for direct and indirect previously obtained E_g_ [51], boosting its potential catalytic activity into the visible range and making synthesized indium oxide nanoparticles suitable for optoelectronic applications with a narrow gap such as light-emitting diodes (LEDs) and UV photodetectors [52,53].

**Figure 6:**
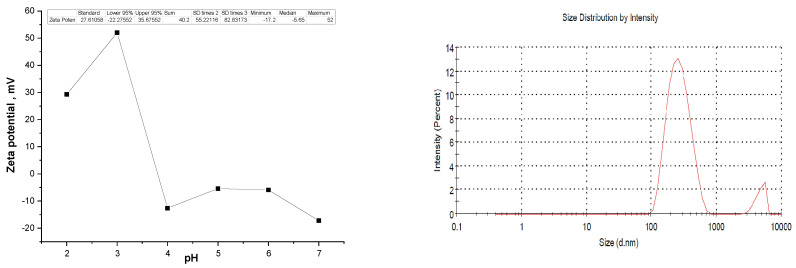
Particle size distribution and zeta potential of the In_2_O_3_-NPs.

Dynamic light scattering (DL), shown in Figure 6a, depicts the zeta potentials of In_2_O_3_ at various pH levels. The dispersion stability of particles in colloids is indicated by the zeta potential. The zeta potentials of the indium oxide were measured at 29.3, 52, −12.6, −5.4, −5.9, and −17.2 mV at pH values of 2, 3, 4, 5, 6, and 7, respectively. The In_2_O_3_ zeta potential decreased as the pH climbed from 2 to 8. At pH 2, the zeta potential was positive at 29.3 mV, suggesting that the surface of indium oxide becomes positively charged in acidic conditions. At pHs 3 and 4, zeta potentials were positive at 52 mV; these values indicate that the surface charge is still predominantly positive but has increased in magnitude. The decrease in positive charge at pH 4 might be due to a decrease in the protonation of surface groups as the pH increases. The positive charge could arise from protonation of surface hydroxyl groups or other acidic functional groups present during pH optimization (1M NaOH and 1M HCl) on the surface. At pHs 5 and 6, zeta potentials were negative at −5.4 mV and −5.9 mV, respectively. This suggests that the surface charge has shifted from being predominantly positive to slightly negative. At these pH values, the hydroxyl groups on the surface may undergo deprotonation, resulting in a negatively charged surface. The zeta potential was more negative at −17.2 mV. This indicates a further increase in the negative charge on the surface. At neutral pH, the surface hydroxyl groups are most likely deprotonated, resulting in a greater proportion of negatively charged sites. These findings indicate that particle aggregation will occur under acidic conditions. Above pH 4, the NPs’ surfaces will be negatively charged, so interaction with positively charged particles will be more favorable. Nanoparticle aggregation state is a function of zeta potential values: if the values decrease (approaching zero), the electrostatic repulsion decreases as well [53,54]. As a result, the In_2_O_3_-NPs become more prone to aggregate, as the attractive forces between them (van der Waals forces, for example) can overcome the weak repulsive forces. The zeta potential of In_2_O_3_-NPs is often influenced by the pH of the surrounding medium. Acidic pH values can lead to the smallest potential zeta values close to zero. At these pH conditions, the surface chemistry of the nanoparticles may change, affecting the balance between attractive and repulsive forces. The altered surface chemistry can contribute to enhanced aggregation tendencies. In addition, potential zeta values close to zero can promote the formation of bridges or connections between nanoparticles. Ions [Na^+^], [OH^−^], [NO_3_^−^], and [H^+^] in the surrounding medium can adsorb onto the nanoparticle surfaces, creating bridges that bring particles closer together. These bridges can enhance the attractive forces and facilitate the aggregation process [55].

Figure 6b shows the particle size distribution of In_2_O_3_-NPs agglomerations and clusters in aqueous media, with an average size around 280 nm.

### 3.2. Effect of pH, Time, and Terbium Concentration on Adsorbents’ Removal Efficiency

**Figure 7:**
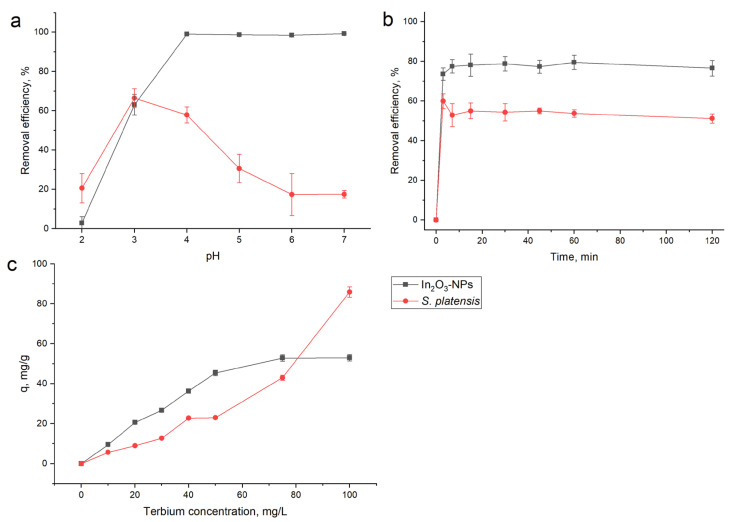
Effect of (**a**) pH, (**b**) time, and (**c**) concentration on terbium removal by In_2_O_3_-NPs and spirulina biomass.

Due to its influence on the chemical speciation of the metal ions in the solution and the ionization of functional groups on the adsorbent surface, the pH is a crucial factor in the adsorption process [54]. To assess the effect of the pH on terbium ion removal, experiments were performed at pH values in the range of 2.0–7.0. At pH 2.0, for both adsorbents, the efficiency of terbium ions removal was low: 2.8% for In_2_O_3_-NPs and 20.5% for spirulina biomass (Figure 7a). Low adsorption can be explained by the competition of hydrogen ions with terbium ions for binding sites on the surface of adsorbents [55]. An increase in the pH facilitated terbium ion removal, and in the case of In_2_O_3_-NPs at a pH range of 4.0–7.0, removal efficiency reached 98–99%. The high efficiency of terbium removal at the mentioned pH values can be associated with its ions binding to OH groups. Following the addition of OH groups to the solution (in the present study due to use of NaOH), cationic [Tb(OH)(H_2_O)_7_]^2+^, [Tb(OH)_2_(H_2_O)_6_]^+^, and neutral [Tb(OH)_3_(H_2_O)_5_] hydroxo complexes can be formed. Thus, it can be suggested that In_2_O_3_-NPs adsorb terbium that is present in the solution as cationic or neutral hydroxo complexes [56]. It has previously been reported that hydrolyzed cationic and even neutral complexes of REEs are priority species for sorption on zeolites and linoptilolites [56]. In the case of spirulina biomass, a maximum terbium removal of 66% was attained at pH 3.0, when the biomass surface became negatively charged (Figure 1), leading to an enhanced adsorption. The obtained results are in line with other research studying the adsorption of REEs onto spirulina biomass [57]. A further increase in the pH values resulted firstly in the slight decrease in the terbium removal at pH 4.0 (57%), and then, it was significantly reduced and at pH 6.0–7.0 amounted only to 17%. Terbium, as other REEs, exists in the form Tb^3+^ at pH < 4.0, while at higher pH values, their species including Tb(OH)_2_^+^ and Tb(OH)_3_ are formed [58]. The decrease in terbium removal at pH > 4 can be associated with the increase in the hydroxide ligands due to the use of NaOH for the adjustment of pH, which resulted in the formation of terbium species for which spirulina biomass possesses a low adsorption capacity. Thus, it can be suggested that different mechanisms are involved in terbium adsorption for the two studied adsorbents.

A pH of 8.5 was found to be optimal for terbium removal via Transcarpathian clinop-tilolite [56]. Maximum terbium removal using 1-(2-pyridylazo)-2-naphthol (PAN)-doped hybrid silica was attained at pH 4.0 [59], via Ca(Ⅱ)-modified garlic peels at pH 3.5 [60], and via activated carbon at pH 5.0 [61]. Thus, the subsequent adsorption experiments were carried out at pH 4.0 for In_2_O_3_-NPs and pH 3.0 for spirulina biomass.

The effect of contact time on removal was studied in order to define the equilibrium point at which the maximum adsorption capacity could be achieved and to explain the kinetics of the process [62]. The influence of the time on the removal of terbium ions is illustrated in Figure 7b. For both adsorbents, the removal efficiency sharply increased in the first 3 min of sorbent interaction with the sorbate, reaching 74% removal for In_2_O_3_-NPs and 60% for spirulina biomass. The fast adsorption in the first minute of interaction becomes almost insignificant in the next 120 min of reaction, and so, equilibrium was attained in a very short time, a fact that is very important for the industrial applications of adsorbents. The enhanced sorption of terbium in the rapid phase of interaction can be explained by the availability of a large number of well-aligned binding sites on the adsorbents surface, and their saturation leads to the establishment of equilibrium [63].

With the increase in the initial concentration of terbium from 10 to 100 mg/L, the amount of elements adsorbed increased from 5.7 to 85.8 mg/g for spirulina biomass and from 9.4 to 60 mg/g for In_2_O_3_-NPs (Figure 7c). The increase in terbium adsorption with the increase in its ion concentration is explained by a more frequent interaction between metal ions and adsorbents [64].

### 3.3. Equilibrium and Kinetics of the Terbium Adsorption Process

Langmuir, Freundlich, and Temkin isotherm models were applied to describe the adsorption equilibrium. The Langmuir model assumes monolayer adsorption onto a surface with a finite number of identical sites and is expressed by Equation (6) [27]:

(6) $$q_{m}=\frac{q_{m\;}{\mathrm{bC}}_{e}}{1+{\mathrm{bC}}_{e}}$$

The Freundlich isotherm model, which is empirical in nature, is applied to describe adsorption on heterogeneous surfaces [65]:

(7) $$q_{m}=K_{F}{\mathrm{Ce}}^{\frac{1}{n}}$$

The Temkin isotherm model assumes that during sorbent interaction with sorbate, the adsorption heat of all molecules in the layer decreases linearly with coverage due to adsorbent–adsorbate interactions, and that adsorption is characterized by a uniform distribution of binding energies [25,66], Equation (8):

(8) $$q_{e}=\frac{\mathrm{RT}}{b_{T}}\ln\left(a_{T}C_{e}\right)$$

$$B=\frac{\mathrm{RT}}{b}$$

Langmuir constants q_m_ (mg/g) and b (L/mg) relate to the energy of adsorption and maximum adsorption capacity, respectively; K_F_ (mg/g) and n are Freundlich constants which correspond to adsorption capacity and adsorption intensity, respectively; b_T_ (J/mol) is the Temkin constant related to the heat of adsorption, a_T_ (L/g) is the constant of equilibrium binding, R is the universal gas constant (8.314 J K^−1^ mol^−1^), and T is the temperature (K).

**Figure 8:**
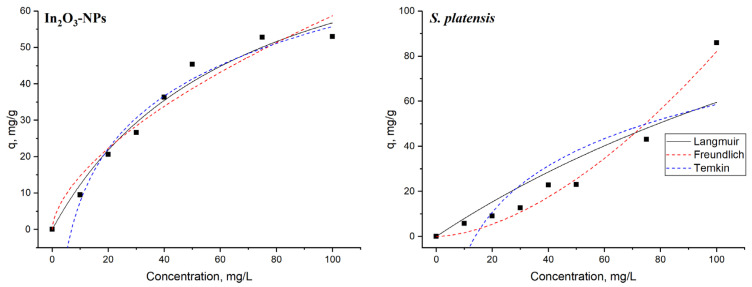
The adsorption isotherms for terbium ions adsorption onto In_2_O_3_-NPs and spirulina biomass (time 1 h, temperature 22 °C, adsorbent dosage 0.1 g).

The non-linear equilibrium models’ plots are shown in Figure 8, while the equilibrium models’ constants and correlation coefficients are presented in Table 2. 

According to the correlation coefficient values, the Langmuir model was the most applicable for the explanation of the terbium adsorption onto In_2_O_3_-NPs. The model suggests that once a site is occupied, no more sorption can occur there [65]. In the case of spirulina biomass, terbium adsorption obeys the Freundlich model with R^2^ = 0.97. That model, which is applicable to the description of adsorption on heterogeneous surfaces, assumes that once the sorption centers are saturated, the sorption energy will rapidly decline [67]. The n value for In_2_O_3_-NPs was higher than 1.0, indicating favorable conditions for sorption, while for spirulina biomass, the value was lower than 1.0, which implies that the adsorption process is related to a chemical process [65]. The highest values of q_m_ amounted to 94.7 mg/g for In_2_O_3_-NPs and 212 mg/g for spirulina biomass. For both adsorbents, the experimentally obtained adsorption capacity was lower than values obtained theoretically, suggesting that the surface of the adsorbents during terbium ions adsorption was not fully covered [68]. Based on the correlation coefficient values, the Temkin model also fit with the adsorption of terbium ions into In_2_O_3_-NPs. Thus, electrostatic interaction is one of the mechanisms of terbium adsorption onto nanoadsorbents [69]. The Temkin constant (b) values related to the heat of adsorption constituted 21 kJ/mol for In_2_O_3_-NPs and 30 kJ/mol for spirulina biomass. The maximum binding energy for In_2_O_3_-NPs was two times higher than for the spirulina biomass (Table 2). It is known that the typical range of bonding energy for an ion-exchange mechanism is 8–16 kJ/mol [64].

The maximum adsorption capacity computed from the Langmuir model was compared with values reported in the literature for other types of adsorbents (Table 3). Values obtained in the present study are among the highest.

In order to explain the adsorption process, Lagergren’s pseudo-first-order and pseudo-second-order models and the Elovich kinetic model were applied. The pseudo-first-order model suggests one-site-occupancy adsorption [68]:

(9) $$q_{t}={\;q}_{e\;}\left(1-e^{-k_{1}t}\right)$$

The pseudo-second-order model is suitable for the description of the chemical adsorption, which involves a chemical adsorption between the negatively charged surface and metal ions:

(10) $$q=\frac{q_{e}^{2}k_{2}t}{1+q_{e}k_{2}t}$$

The Elovich model is used to describe chemical adsorption on heterogeneous surfaces. The model assumes that the rate of biosorption decreases exponentially with an increase in the amount of adsorbate [70]:

(11) $$q_{t\;}=\frac{1}{\beta }\ln\left(1+\alpha \beta t\right)$$

where q_t_ is the amount of adsorbed metal (mg/g) at time t, (mg/g); k_1_ (1/min) is the rate constant of the first-order adsorption; k_2_ (g/mg·min) is the rate constant of the second-order adsorption; and α (g/mg∙min) and β (g/mg) are Elovich model constants representing the initial reaction rate and desorption energy obtained from the Elovich equation, respectively.

**Figure 9:**
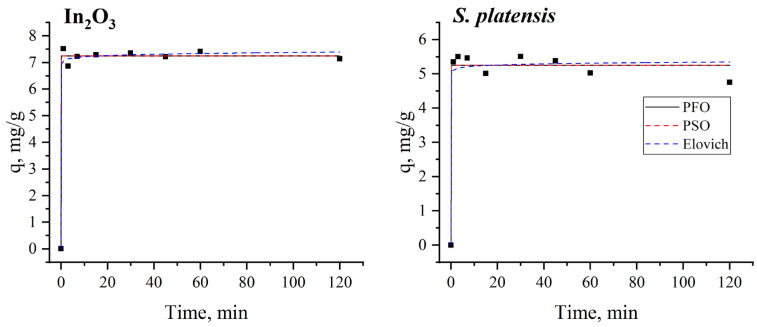
Adsorption kinetics applied to explain terbium ions adsorption onto In_2_O_3_-NPs and spirulina biomass (C_i,Tb_ 10 mg/L, temperature 22 °C, adsorbent dosage 0.1 g).

The non-linear fitting of the experimental results is presented in Figure 9. Experimentally calculated adsorption parameters and correlation coefficients are listed in Table 4.

According to the correlation coefficient values, the pseudo-second-order and Elovich models were most applicable to describing terbium adsorption onto In_2_O_3_-NPs, suggesting a chemical sorption [71]. It is suggested that the adsorption of terbium ions may consist of two phases: first, terbium ions are transferred to the binding sites, and in the next stage, the interaction via chemical complexation or ion exchange takes place [67]. In the case of spirulina biomass, the pseudo-first-order and pseudo-second-order models were adequate for describing terbium removal. A good correlation between experimental and calculated adsorption capacity was obtained for both models. The applicability of the pseudo-first-order model shows that, for terbium ions, adsorption onto spirulina biomass occurs exclusively onto one site per ion, while the pseudo-second-order model indicates that the sorption is chemical in nature [72].

### 3.4. Mechanism of Terbium Ions Adsorption

**Figure 10:**
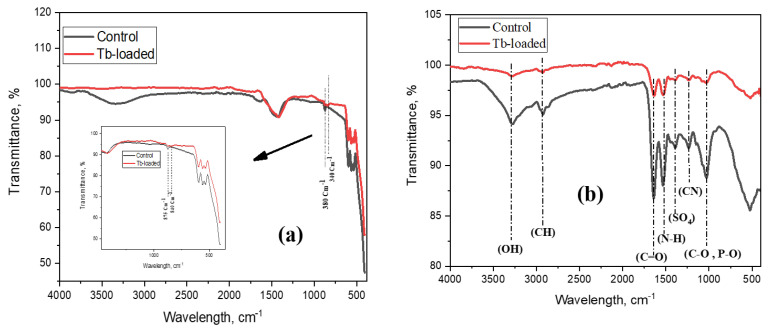
FTIR spectra of (**a**) In_2_O_3_-NPs and (**b**) spirulina biomass before and after terbium ions adsorption.

FTIR spectra of adsorbents were analyzed before and after the adsorption of terbium ions in order to reveal the involvement of functional groups in the ions’ removal. In the spectrum of In_2_O_3_-NPs (Figure 10a), peaks positioned at the wavenumbers 601, 562, and 532 cm^−1^, which correspond to In-coordinated oxygen (In-O), indium-to-indium stretching (In-In), and the stretching manner of the two atoms of indium when mutually coordinated with oxygen (In-O-In), respectively, were observed [73]. It can be clearly seen that the intensity of the bands at 3180−3500 cm^−1^, which correspond to the stretching vibration of hydrogen bonds due to the abundance of hydroxyl groups of moisture that are adsorbed at the In_2_O_3_-NPs’ surface, was greatly reduced after terbium adsorption [41,74]. In the case of the In_2_O_3_-NPs’ spectrum after terbium adsorption, a new band at 840 cm^−1^ can be classified as a satellite peak that appears on the low wavenumber side of the main In-O stretching band at 870 cm^−1^. The band may arise due to the presence of defects, impurities, or other structural variations in the In_2_O_3_ lattice, which can affect the bonding and symmetry of the In-O units due to adsorption collisions and Tb^+3^ agglutinations [41,74,75].

In the spectrum of control for spirulina biomass (Figure 10b), the band at 3280 cm^−1^ is assigned to the stretching of O-H groups and the one at 2920 and 2851cm^−1^ to asymmetric C-H stretching. The band at 1640 cm^−1^ could be assigned to C=O, present in the lipids of A platensis [76]. The strong band at 1541 cm^−1^ is attributed to the N-H bending of amide groups that are present in cyanobacteria [77]. The shoulder at 1450 and the band at 1390 cm^−1^ could be assigned to sulfates groups, while the band at 1236 cm^−1^ is assigned to the C-N vibrations. The band at 1022 cm^−1^ could be assigned to the C-O stretching of carbohydrates or lipids as well as to the P-O bonds of phosphates groups [78]. In Tb-loaded spirulina biomass, the intensities of the bands corresponding to all function groups have been diminished, which can be associated with the terbium ion binding to functional groups, which results in the occurrence of bond stretching to a lesser degree [79]. Ion-exchange is another possible mechanism of terbium ions adsorption onto spirulina biomass. Thus, it was shown previously that Dy adsorption onto spirulina biomass was accompanied by the decrease in content of Mg, Ca, Cl, and Mn in the biomass [80].

## 4. Conclusions

The nanoadsorbent In_2_O_3_ and the biological sorbent *Arthrospira platensis* showed high adsorption capacity for terbium ions removal. A maximum terbium removal of 98–99% using In_2_O_3_ NPs was attained at pH range 4.0–6.0, while the spirulina biomass achieved better removal of metal ions at pH 3.0 (66%). For both adsorbents, terbium removal was a two-step process, with maximum removal in the first 3 min of interaction and rapid achievement of equilibrium. Terbium adsorption onto In_2_O_3_ NPs was best described applying the Langmuir model, while the Freundlich model was more applicable for spirulina biomass. The maximum theoretical adsorption capacity of spirulina biomass (212 mg/g) exceeds the value obtained for In_2_O_3_ NPs (94.3 mg/g). The kinetics of terbium adsorption onto In_2_O_3_ NPs fit the pseudo-second-order and Elovich models, while for spirulina biomass, it fit the pseudo-second-order and pseudo-first-order models. The applicability of the aforementioned models indicates a significant role of chemisorption in the removal of terbium ions, and the results of FTIR analysis support this interpretation. The studied adsorbents have good potential for the recovery of terbium ions.

## Figures and Tables

**Table 1:** The crystal structure parameters of precursors and In_2_O_3_-NPs.

Sample Name	(hkl)	d-Spacing	βD (rad)	D (Scherrer eq.) (nm)	δ (nm 2)	ε%
Precursor	2 2 0	0.4	0.1899	0.743	1.8	24.2
In_2_O_3_-NPs	2 2 2	0.29	0.005031	28.5	0.00122	0.4582

**Table 2:** The constants and correlation coefficients of the adsorption isotherms, applied to describe terbium adsorption.

	Langmuir			Freundlich			Temkin		
	q_m,_ mg/g	b, L/mg	R^2^	K_f_, mg/g	n	R^2^	a_T_, L/g	b_T_, J/mol	R^2^
In_2_O_3_-NPs	94.7 ± 13.6	0.01 ± 0.004	0.98	3.69 ± 0.3	1.66 ± 0.24	0.95	0.14 ± 0.02	116.7 ± 8.8	0.97
*S. platensis*	212 ± 35	0.004 ± 0.008	0.81	0.03 ± 0.003	0.59 ± 0.06	0.97	0.072 ± 0.02	82.1 ± 22.3	0.73

**Table 3:** Maximum sorption capacity obtained for analyzed adsorbents compared with data from the literature.

Sorbent	q, mg/g	Reference
*Spirulina platensis*	212	Present work
In_2_O_3_-NPs	94.7	Present work
natural clinoptilolite	1.67–3.85	[56]
1-(2-pyridylazo)-2-naphthol (PAN)-doped hybrid silica	123.6	[59]
Ca(Ⅱ)-modified garlic peels	0.06 μg/g	[60]
Activated Carbon	14.9	[61]

**Table 4:** The constants and correlation coefficients of the kinetic models.

	Pseudo-First-Order			Pseudo-Second-Order			Elovich		
	q_e_, mg/g	k_1_, 1/min	R^2^	q_e_, mg/g	k_2_, g/mg·min	R^2^	α, g/mg∙min	Β, g/mg	R^2^
In_2_O_3_	7.27 ± 0.03	0.96 ± 0.07	0.97	7.32 ± 0.05	0.78 ± 0.24	0.99	2.64 × 10^38^ ± 1.50 × 10^34^	13.0 ± 8.03	0.99
*S. platensis*	5.23 ± 0.11	6.5 ± 0.07	0.98	5.23 ± 0.11	0.75±0.17	0.98	2.22 × 10^43^ ± 1.73 × 10^40^	20.3 ± 4.8	0.97

## Data Availability

Not applicable.

## Supplementary Materials

The following supporting information can be downloaded at: https://www.mdpi.com/article/10.3390/nano13192698/s1, Figure S1: D-spacing analysis of indium oxide nanoparticles attached to reference card (01-089-4595); Figure S2: Comparative peak list (2θ) of synthesized In_2_O_3_-NPs (orange color) related to standard In_2_O_3_ reference card (01-089-4595) (blue color); Table S1: D-spacing analysis of indium oxide nanoparticles. Figure S3. Comparative peak list (2θ) of synthesized precursor (orange color) related to standard different references cards. Supplementary File S1: the raw data of zeta potential measurements.
