# Supplementary material for: Terbium Removal from Aqueous Solutions Using a In2O3 Nanoadsorbent and Arthrospira platensis Biomass
Source: Nanomaterials (Basel). 2023 Oct 3;13(19):2698. doi: 10.3390/nano13192698 (PMC10574616; doi:10.3390/nano13192698)
Supplement: Supplementary file 1 [file nanomaterials-13-02698-s001.zip › nanomaterials-2620414-supplementary.pdf]

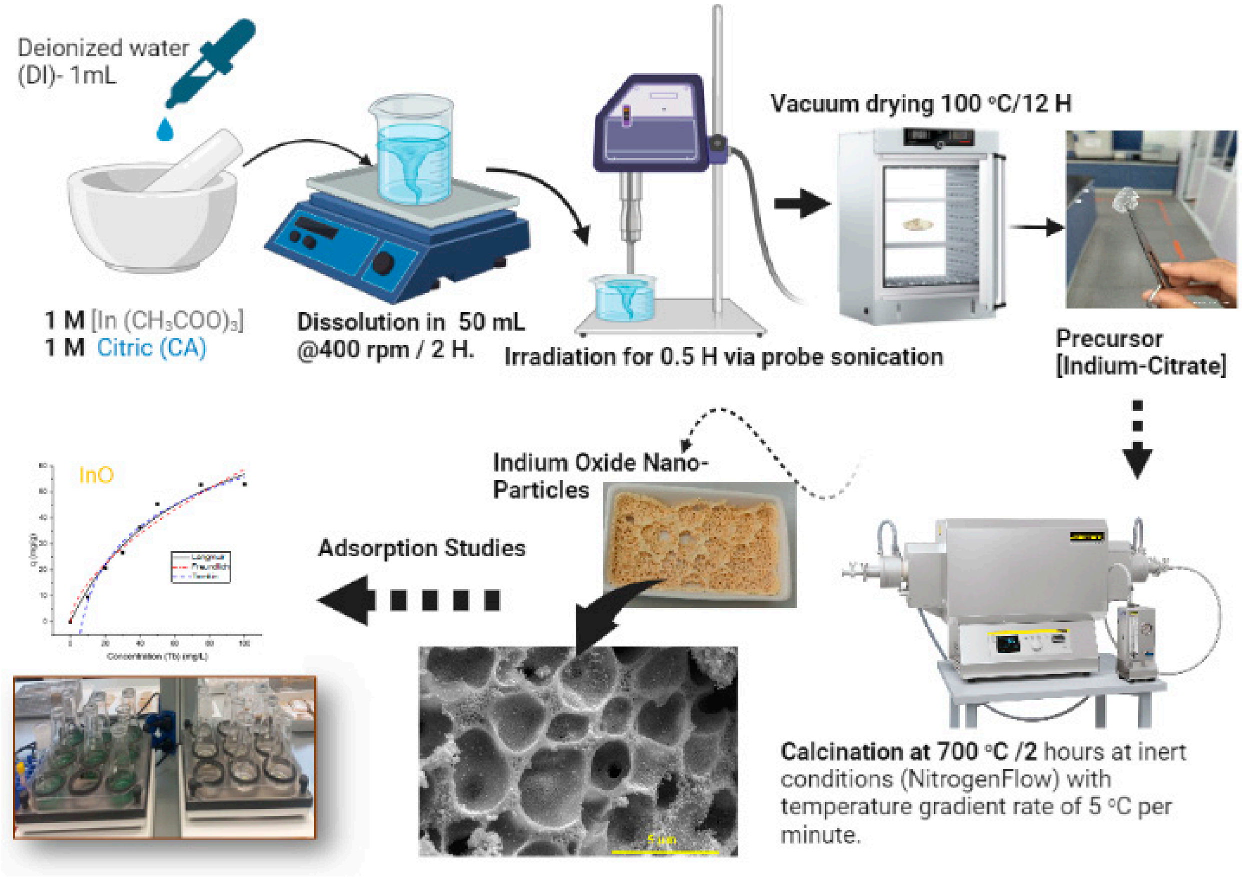

Figure SC. Schematic diagram of Indium Oxide Nanoparticle's synthesis.

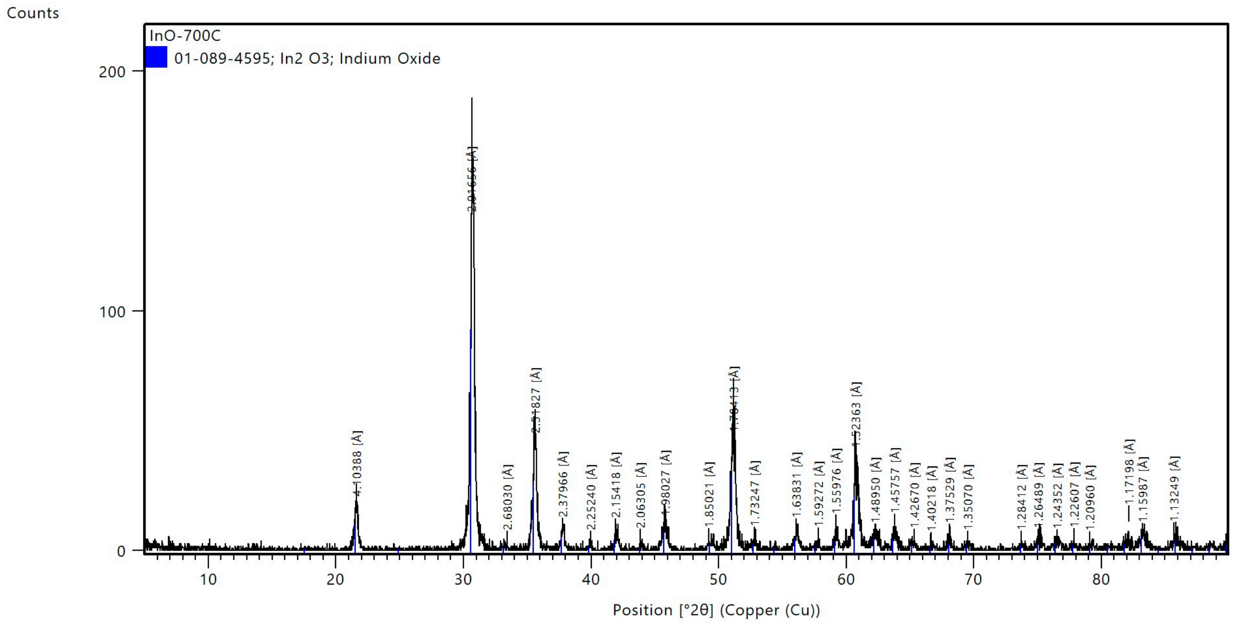

Figure S1. D-Spacing analysis of Indium Oxide Nanoparticles attached to reference card (01-089-4595).

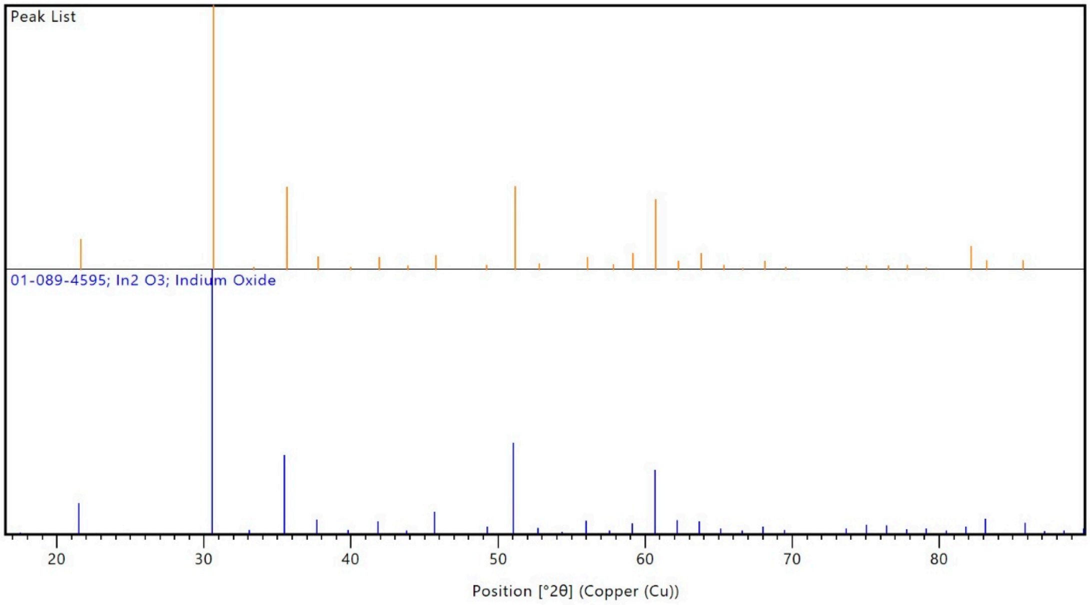

Figure S2. Comparative Peak list (2θ) of synthesized In_2O_3-NPs (orange color) related to standard In_2O_3 reference card (01-089-4595) (blue color).

Table S1. D-Spacing analysis of Indium Oxide Nanoparticles.

Rel. Int. [%]	Height [cps]	Height [cts]	d-spacing [Å]	Pos. [°2θ]
11.51	30.84	15.42	4.10388	**21.6372**
100	267.89	133.95	2.91656	**30.6285**
1.06	2.84	1.42	2.6803	**33.4039**
31.09	83.29	41.65	2.51827	**35.6227**
4.9	13.14	6.57	2.37966	**37.7738**
1.01	2.71	1.35	2.2524	**39.9964**
4.77	12.78	6.39	2.15418	**41.9038**
1.46	3.92	1.96	2.06305	**43.8484**
5.43	14.54	7.27	1.98027	**45.7831**
1.8	4.82	2.41	1.85021	**49.2062**
31.47	84.3	42.15	1.78413	**51.1573**
2.22	5.94	2.97	1.73247	**52.7986**
4.74	12.71	6.35	1.63831	**56.0918**
1.93	5.16	2.58	1.59272	**57.8464**
6.07	16.27	8.14	1.55976	**59.1891**
26.63	71.33	35.66	1.52363	**60.7381**
3.23	8.64	4.32	1.4895	**62.2831**
6.23	16.7	8.35	1.45757	**63.8058**
1.6	4.27	2.14	1.4267	**65.3557**
0.56	1.5	0.75	1.40218	**66.646**
3.22	8.63	4.32	1.37529	**68.1254**
0.94	2.53	1.26	1.3507	**69.5417**
0.94	2.52	1.26	1.28412	**73.7206**
1.54	4.12	2.06	1.26489	**75.0321**
1.41	3.77	1.89	1.24352	**76.5523**
1.71	4.57	2.29	1.22607	**77.8446**
0.78	2.1	1.05	1.2096	**79.1102**
8.74	23.42	11.71	1.17198	**82.1829**
3.45	9.24	4.62	1.15987	**83.2302**
3.54	9.48	4.74	1.13249	**85.7162**

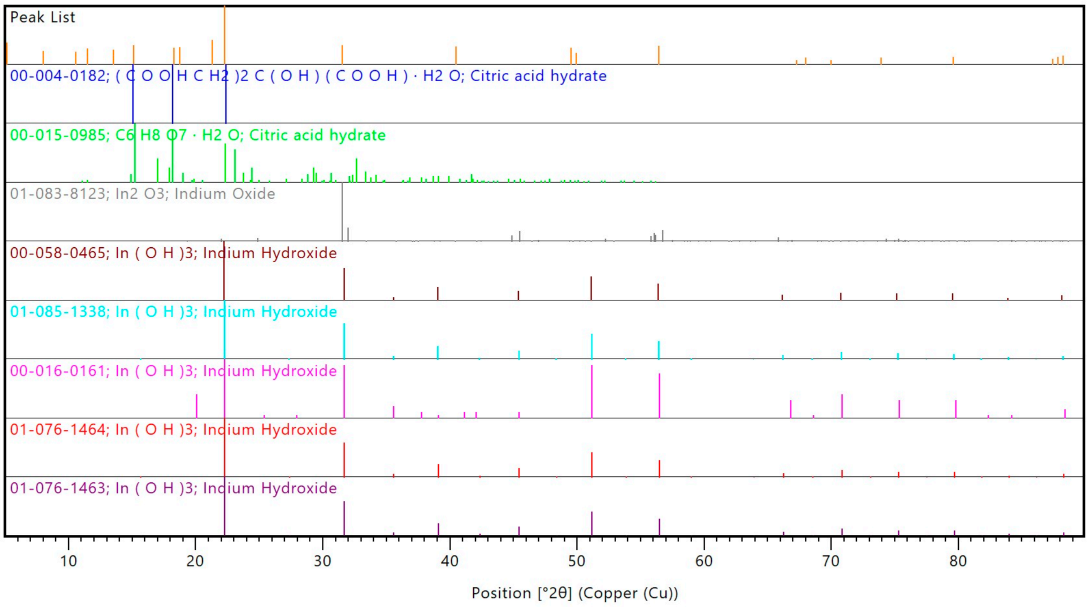

Figure S3. Comparative Peak list (2θ) of synthesized Precursor (orange color) related to standard different references cards.
