# Supplementary material for: Terbium Removal from Aqueous Solutions Using a In2O3 Nanoadsorbent and Arthrospira platensis Biomass
Source: Nanomaterials (Basel). 2023 Oct 3;13(19):2698. doi: 10.3390/nano13192698 (PMC10574616; doi:10.3390/nano13192698)

# Zeta Potential Report

*v2.3*

Malvern Instruments Ltd - © Copyright 2008

## Sample Details

**Sample Name:** 4 1

**SOP Name:** mansettings.nano

**General Notes:**

| | | | |
|---|---|---|---|
| **File Name:** | Dr. Islam.dts | **Dispersant Name:** | Water |
| **Record Number:** | 15 | **Dispersant RI:** | 1.330 |
| **Date and Time:** | Thursday, June 22, 2023 7:38:11 | **Viscosity (cP):** | 0.8872 |
| | | **Dispersant Dielectric Constant:** | 78.5 |

## System

| | | | |
|---|---|---|---|
| **Temperature (°C):** | 25.1 | **Zeta Runs:** | 12 |
| **Count Rate (kcps):** | 22.2 | **Measurement Position (mm):** | 2.00 |
| **Cell Description:** | Clear disposable zeta cell | **Attenuator:** | 7 |

## Results

| | | | Mean (mV) | Area (%) | St Dev (mV) |
|---|---|---|---|---|---|
| **Zeta Potential (mV):** | -12.6 | **Peak 1:** | -12.6 | 100.0 | 5.78 |
| **Zeta Deviation (mV):** | 5.78 | **Peak 2:** | 0.00 | 0.0 | 0.00 |
| **Conductivity (mS/cm):** | 0.0360 | **Peak 3:** | 0.00 | 0.0 | 0.00 |
| **Result quality :** | Good | | | | |

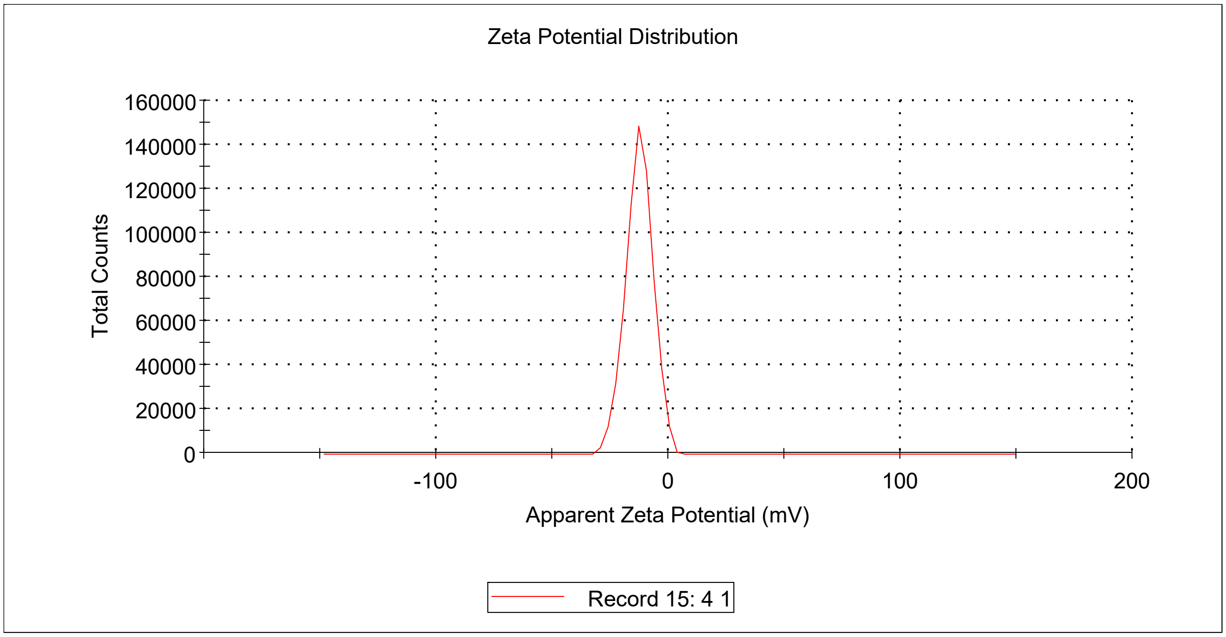

Supplement: Supplementary file 1 [file nanomaterials-13-02698-s001.zip › Supplemnetary File S1/Zeta pH 4 (22-06-2023).pdf]
