# Supplementary material for: Terbium Removal from Aqueous Solutions Using a In2O3 Nanoadsorbent and Arthrospira platensis Biomass
Source: Nanomaterials (Basel). 2023 Oct 3;13(19):2698. doi: 10.3390/nano13192698 (PMC10574616; doi:10.3390/nano13192698)

Zeta Potential Report

v2.3

Malvern Instruments Ltd - © Copyright 2008

Sample Details

Sample Name: 5 1

SOP Name: mansettings.nano

General Notes:

**File Name:**	Dr. Islam.dts	**Dispersant Name:**	Water
**Record Number:**	16	**Dispersant RI:**	1.330
**Date and Time:**	Thursday, June 22, 2023 7:41:50	**Viscosity (cP):**	0.8872
		**Dispersant Dielectric Constant:**	78.5

System

**Temperature (°C):**	24.9	**Zeta Runs:**	12
**Count Rate (kcps):**	315.7	**Measurement Position (mm):**	2.00
**Cell Description:**	Clear disposable zeta cell	**Attenuator:**	8

Results

			Mean (mV)	Area (%)	St Dev (mV)
**Zeta Potential (mV):**	-5.46	**Peak 1:**	-5.46	100.0	5.17
**Zeta Deviation (mV):**	5.17	**Peak 2:**	0.00	0.0	0.00
**Conductivity (mS/cm):**	0.00853	**Peak 3:**	0.00	0.0	0.00
**Result quality :**	Good				

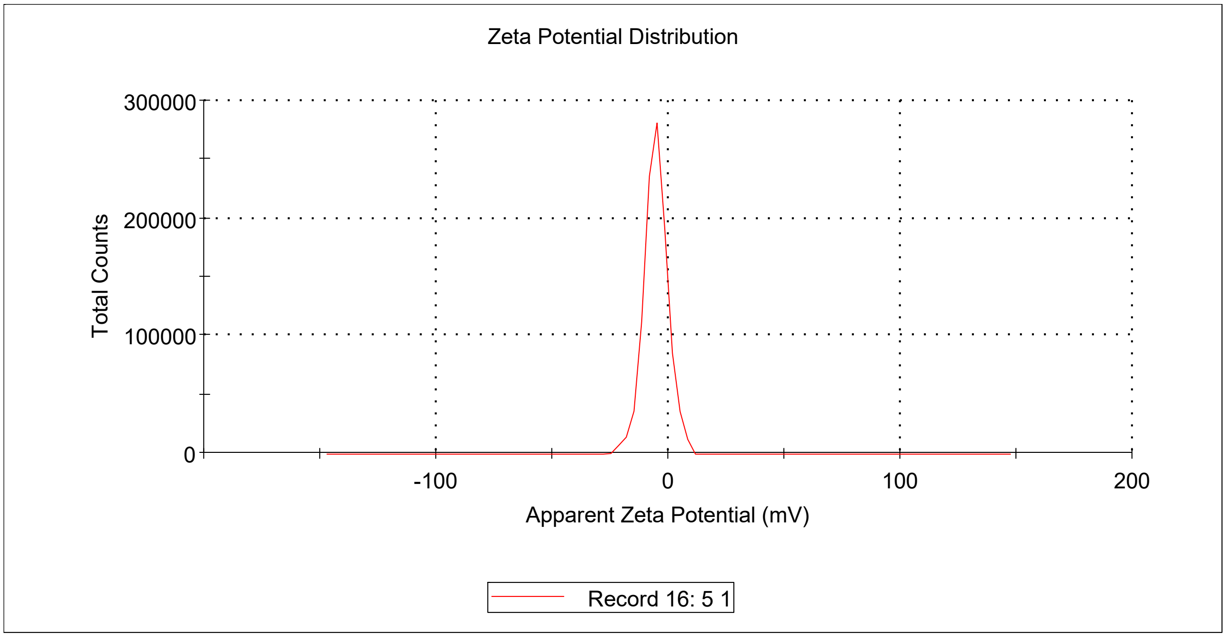

Supplement: Supplementary file 1 [file nanomaterials-13-02698-s001.zip › Supplemnetary File S1/Zeta pH 5 (22-06-2023).pdf]
